# Catecholamine-associated left ventricular hypertrophy in dogs with hyperadrenocorticism and myxomatous mitral valve disease: a prospective pilot study

**DOI:** 10.4142/jvs.26013

**Published:** 2026-07-23

**Authors:** Hwi Park, Jung-Hyun Kim

**Affiliations:** Department of Veterinary Internal Medicine, College of Veterinary Medicine, Konkuk University, Seoul 05029, Korea.

**Keywords:** Adrenal disease, mitral valve insufficiency, normetanephrine, cardiac remodeling, speckle-tracking echocardiography

## Abstract

**Importance:**

Endogenous Cushing’s syndrome increases the risk of cardiac hypertrophy, dysfunction, and cardiovascular mortality. Given the high prevalence of hyperadrenocorticism (HAC) and myxomatous mitral valve disease (MMVD) in dogs, endocrine-cardiac interactions may influence disease progression but remain poorly characterized.

**Objective:**

To evaluate the effects of concurrent HAC on cardiac remodeling and function in dogs with subclinical MMVD and identify metabolic and neurohormonal markers, particularly catecholamine metabolites, associated with left ventricular hypertrophy and dysfunction.

**Methods:**

Twenty-one client-owned dogs (HAC + MMVD, MMVD-only controls, and healthy controls [each n = 7]) were prospectively enrolled. Echocardiographic variables, including speckle-tracking echocardiography, hematologic and biochemical parameters, and urinary normetanephrine: creatinine ratios, were evaluated at baseline and follow-up.

**Results:**

The HAC + MMVD group showed significantly increased urinary normetanephrine: creatinine ratios than both healthy control group and MMVD-only control group (*p* = 0.011 and *p* = 0.007, respectively). Urinary normetanephrine: creatinine ratio was independently associated with interventricular septum wall thickness in diastole (IVSdN, B = 0.000222, 95% confidence interval [CI], 0.000050 to 0.000393, *p* = 0.026). Δ Urinary normetanephrine: creatinine was positively associated with Δ IVSdN after adjusting for Δ days (B = 0.000308, 95% CI, 0.000108 to 0.000507, *p* = 0.016). Global longitudinal strain (GLS) was lower in the HAC + MMVD group than both the healthy control group and the MMVD-only control group (*p* = 0.001 and *p* = 0.002, respectively). However, GLS was not associated with normetanephrine levels (r = 0.179, 95% CI, −0.698 to 1.000, *p* = 0.720).

**Conclusions and Relevance:**

This exploratory pilot study is the first suggesting a potential neuroendocrine mechanism underlying endocrine-cardiac remodeling in dogs with concurrent HAC and MMVD, whereby increased catecholamine activity may be associated with left ventricular hypertrophy.

## INTRODUCTION

Endogenous Cushing’s syndrome in humans is associated with an increased risk of left ventricular hypertrophy (LVH), cardiac dysfunction, and overall cardiovascular morbidity, even after successful endocrine treatment [1,2,3,4,5]. These cardiovascular complications are primarily attributed to prolonged exposure to excess cortisol, which promotes structural and functional remodeling of the heart [3,5]. Human studies have also demonstrated that cortisol excess can induce metabolic derangements, activate the renin-angiotensin-aldosterone system, and enhance catecholamine sensitivity, further contributing to adverse cardiac outcomes [6].

Hyperadrenocorticism (HAC), the veterinary analog of Cushing’s syndrome, is one of the most prevalent endocrine diseases in dogs [7,8]. Affected dogs often exhibit systemic hypertension, hypercoagulability, obesity, and dyslipidemia, all of which are recognized cardiovascular risk factors [7,9,10,11]. Recent veterinary studies have also reported structural cardiac alterations in dogs with HAC, including concentric LVH and subclinical systolic dysfunction, identified through echocardiography and speckle-tracking imaging [12,13]. However, the mechanisms underlying HAC-induced myocardial remodeling remain poorly understood.

Myxomatous mitral valve disease (MMVD) is the most common cardiac disease in small-breed dogs and is characterized by progressive mitral regurgitation, volume overload, and compact cardiac remodeling [14]. Although a recent prospective study investigated dogs with concurrent MMVD and HAC, HAC did not demonstrate a significant impact on MMVD [15].

Among the proposed mediators, catecholamines and their metabolites have emerged as potential contributors to cardiac hypertrophy and dysfunction [16,17]. In both humans and dogs, catecholamine excess—whether due to endogenous stress, adrenal tumors, or systemic disease—has been associated with myocardial fibrosis, increased wall thickness, and reduced myocardial strain [16,17,18]. Notably, normetanephrine, a metabolite of norepinephrine, can be reliably measured in urine and may serve as a marker of sustained sympathetic activity [19,20]. In canine HAC, urinary normetanephrine levels are frequently elevated and often persist despite medical treatment, suggesting chronic neurohormonal activation [20].

Moreover, metabolic disturbances—such as increased total cholesterol and triglycerides and altered body fat distribution—have also been implicated in cardiovascular remodeling [6,21,22]. Although lipoprotein abnormalities have been extensively studied in humans, veterinary data remain limited [22,23], and their relevance in dogs with HAC and MMVD has not yet been explored.

Given the high prevalence of HAC and MMVD in dogs and the potential for synergistic deleterious cardiovascular effects, a deeper understanding of their interactions is essential. This prospective pilot study investigated the hypothesis that HAC exacerbates cardiac remodeling in dogs with MMVD through catecholamine-mediated and metabolic mechanisms. We employed advanced echocardiographic techniques, including speckle-tracking echocardiography (STE), and analyzed neurohormonal and metabolic biomarkers to identify the putative pathways contributing to myocardial hypertrophy and dysfunction in this comorbid population.

## METHODS

### Study design and animals

Clinical records, blood and urine samples, and echocardiographic data were prospectively collected from client-owned dogs that presented to the Konkuk University Veterinary Medical Teaching Hospital between November 2024 and October 2025. Written informed consent was obtained from all the owners before the inclusion of their dog in the study.

To minimize the influence of confounding factors associated with cardiac disease, an MMVD-only and a healthy control group were included. The dogs were assigned to one of three groups.

**Group 1) HAC + MMVD group:** dogs diagnosed with HAC and concurrent MMVD, classified as American College of Veterinary Internal Medicine (ACVIM) stage B.
**Group 2) MMVD-only control group:** dogs diagnosed with stage B MMVD without clinical or laboratory evidence of HAC.
**Group 3) Healthy control group:** clinically healthy dogs with no evidence of systemic or cardiovascular disease.

The diagnosis of HAC was based on congruent clinical signs (e.g., polyuria, polydipsia, polyphagia, panting, truncal alopecia, pot belly, and muscle weakness), supporting laboratory abnormalities (elevated hepatic enzymes, hyperlipidemia, thrombocytosis, and lymphopenia), abdominal ultrasonographic findings, and at least one positive endocrine test (low-dose dexamethasone suppression test and/or adrenocorticotropic hormone stimulation test) in accordance with the ACVIM consensus guidelines [7].

The diagnosis and staging of MMVD were established according to the ACVIM consensus guidelines [14], and only dogs classified as stage B were included.

Dogs were excluded if they exhibited glycosuria, persistent proteinuria (urine protein creatinine ratio > 0.5 on two separate occasions), recent blood loss, dehydration, or receiving medications affecting cardiovascular parameters (e.g., corticosteroids, levothyroxine, and phenylpropanolamine). Additional exclusion criteria included comorbidities that could influence cardiovascular function, such as IRIS stage ≥ 2 kidney disease, hypothyroidism, diabetes mellitus, neoplasia, pulmonary thromboembolism, heartworm disease, and congenital cardiac abnormalities.

To examine changes in cardiac wall thickness, function, and cardiovascular variables over time, dogs in the HAC + MMVD and MMVD-only control groups were re-evaluated at follow-up visits in alignment with standard cardiology clinical practice. The healthy controls underwent a single baseline assessment.

To reduce the risk of false-positive findings arising from multiple comparisons, variables that showed significant between-group differences were further evaluated for correlations with ventricular wall thickness indices, and their potential associations were additionally assessed using multivariable linear regression and by examining whether changes in these variables paralleled changes in wall thickness indices over time.

### Clinical and laboratory data collection

Signalment and clinical signs were recorded based on owner reports. HAC subtype was classified based on abdominal ultrasonography: bilateral, symmetric adrenal enlargement indicated pituitary-dependent hyperadrenocorticism, whereas unilateral nodular enlargement indicated a functional adrenal tumor [12].

The body condition score (BCS) was assessed on a 9-point scale ranging from very thin (1) to very obese (9), with scores of 4 and 5 considered ideal [24]. Truncal length (TL) was measured from the shoulder to the ischial tuberosity in centimeters (cm). The modified body mass index (MBMI) was calculated as body weight (kg) divided by TL squared in meters (BW/[TL]^2^). Waist circumference (WC) was measured at the level of the fourth lumbar vertebra in a standing position. Ilium wing distance (IWD) was measured between the dorsal iliac crests, and the hock-to-stifle distance (HSD) was measured from the stifle joint to the hock. WC was normalized to IWD, TL, and HSD to generate the following anthropometric ratios, WIWDR (WC/IWD), WTLR (WC/TL), and WHSDR (WC/HSD). All morphometric measurements were performed as previously described [21].

Systolic blood pressure (SBP), diastolic blood pressure, mean blood pressure, and heart rate (HR) were measured using a high-definition oscillometric device (Cardell Insight Veterinary Monitor, INSIGHT-X000; Midmark Corp., USA) after a 10–15-min acclimation period. Five consecutive consistent readings were obtained; the highest and lowest values were excluded and the remaining three were averaged. Dogs with SBP ≥ 150 mmHg were classified as hypertensive [10].

For laboratory analyses, 4 mL of blood was drawn from the jugular vein. Two milliliters were used for complete blood count, serum biochemistry, and coagulation testing (e.g., D-dimer and thromboelastography [TEG® 5000 Hemostasis Analyzer; Haemonetics Corp., USA]). The remaining 2 mL was placed in a serum separator tube, allowed to clot for 15 min, and centrifuged at 3,000 rpm for 15 min. Approximately 1 mL of the serum was sent to IDEXX Korea for lipid profile analysis.

The owners provided 5 mL of voided urine collected at home under low-stress conditions, refrigerated it and transported it to the clinic on ice. Upon receipt, the samples were sent to an external laboratory (Greenvet, Korea) for urinary metanephrine analysis.

### Echocardiographic evaluation

#### Conventional echocardiography

All echocardiographic examinations were performed using a single ultrasound unit (EPIQ 7; Philips Ultrasound LLC, USA) equipped with a 3.0–8.0 MHz phased-array transducer and ECG monitoring. The scans were conducted by an experienced veterinarian (H-P) following a standardized procedure.

The measured parameters included left atrial (LA) diameter, aortic root diameter (Ao), LA/Ao ratio, interventricular septal (IVS) thickness, left ventricular free wall (LVFW) thickness, and left ventricular internal dimensions at end-diastole (LVIDd) and end-systole (LVIDs). Functional indices included fractional shortening (FS), ejection fraction (EF), mitral inflow velocities (E, A), E/A ratio, tissue Doppler E’, E/E’ ratio, Tei index, and isovolumic relaxation time. The left ventricle (LV) dimensions were normalized to body weight according to established allometric equations [25].

#### STE

Two-dimensional speckle-tracking echocardiography (2D-STE) was performed using the apical four-chamber view with a frame rate > 50 fps. Two cardiac cycles were acquired and analyzed offline using an Ultrasound Workspace (Philips Ultrasound LLC).

The endocardial borders were manually traced, and the LV was divided into six segments (basal, middle, and apical regions of the septal and lateral walls; Fig. 1A). Segmental peak longitudinal strain (LS) values were obtained from the strain–time curves for each segment (Fig. 1B), and the global longitudinal strain (GLS) was automatically calculated as the average of the peak systolic LS values derived from all six segments (Fig. 1C). The analyses were conducted by a single investigator who was blinded to the group assignments. Although strains are expressed as negative values, changes in LS are typically interpreted using their absolute magnitudes, as recommended by the Task Force on the Standardization of Deformation Imaging [26].

**Fig. 1:**
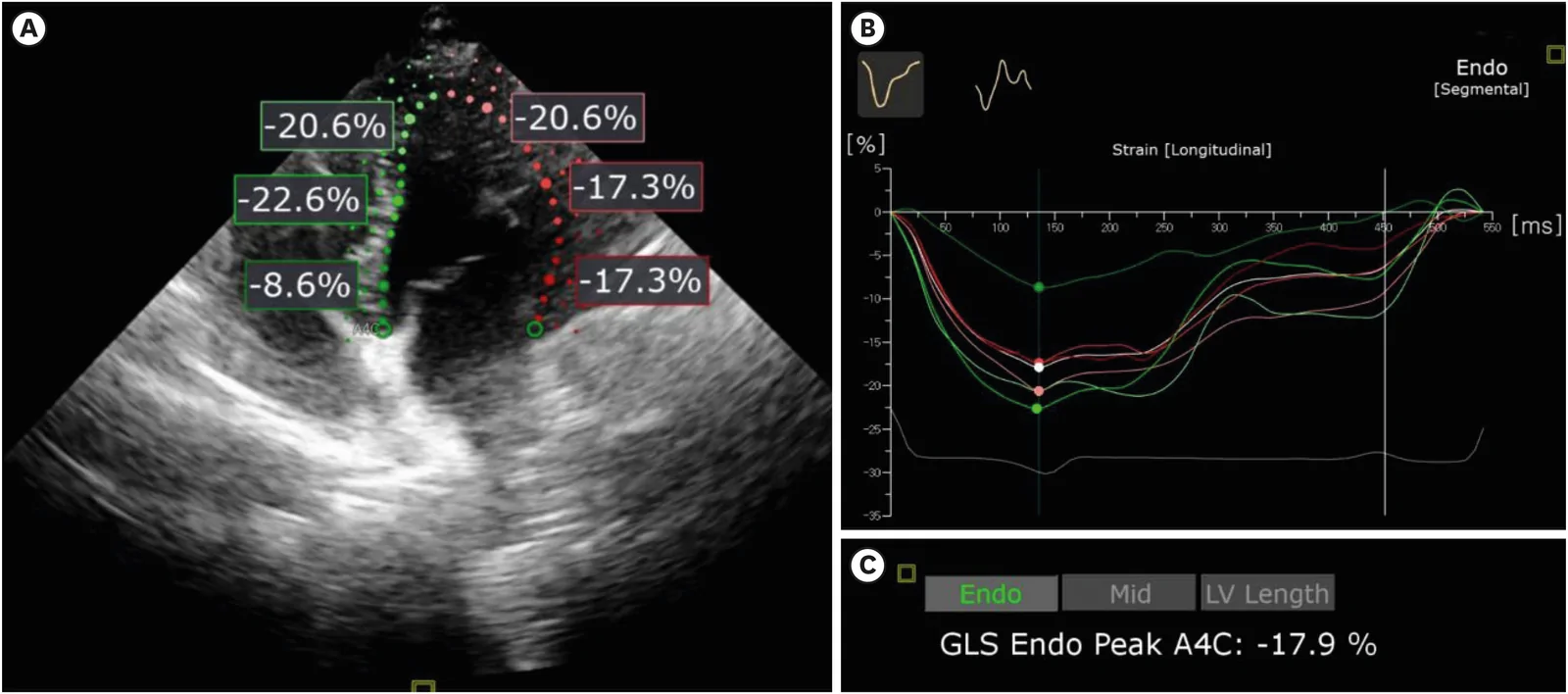
Apical four-chamber speckle-tracking echocardiography. (A) Endocardial border tracking and six-segment LV segmentation at end-diastole; segmental peak longitudinal strain values are labeled clockwise (basal septal −8.6%, mid septal −22.6%, apical septal −20.6%, apical lateral −20.6%, mid lateral −17.3%, basal lateral −17.3%). (B) Strain-time curves for the six segments; each curve’s peak corresponds to the segmental values in (A). (C) The automatically calculated peak GLS is −17.9%. LV, left ventricle; GLS, global longitudinal strain.

### Statistical analysis

All statistical analyses were conducted using SPSS software (version 26.0; IBM Corp., USA). To minimize potential confounding from underlying cardiac diseases, the study group was compared separately with the healthy and MMVD-only control groups. Given the limited sample size, nonparametric statistical methods were used.

Continuous variables were compared using the Mann–Whitney *U* test. Pairwise comparisons were made between the HAC + MMVD and control groups (MMVD only and healthy). Categorical variables were analyzed using Pearson’s χ^2^ or Fisher’s exact tests when the expected frequency in > 20% of the cells was < 5. Echocardiographic variables were analyzed using the mean of three consecutive measurements.

Correlations between continuous and categorical variables were assessed using Spearman’s rank correlation for binary variables and the Kruskal–Wallis test for multi-level categorical variables (≥ 3 categories).

In the HAC + MMVD group, multivariable linear regression was used to identify predictors of left ventricular wall thickness (IVSdN and LVFWdN) and GLS, including variables found to be significant in the univariate analyses (Mann–Whitney *U* or Spearman’s rank correlations). Time-related changes were modeled using change scores (Δ), and the number of days between visits (Δdays) was included as a covariate.

Intra- and inter-observer variability in GLS was assessed in seven randomly selected dogs using the coefficient of variation (CV). The GLS was measured twice by a single investigator (H-P) and independently by two additional blinded observers. Statistical significance was set at *p* < 0.05. Figures were generated using GraphPad Prism (version 8.0; GraphPad Software, USA).

## RESULTS

### Study population

Initially, nine dogs meeting the criteria for HAC + MMVD were recruited. Subsequently, two dogs were excluded because of proteinuria (n = 1), and hypothyroidism (n = 1). Consequently, seven dogs with HAC and concurrent MMVD (stage B) were included in the HAC + MMVD group. The control group comprised seven age- and sex-matched dogs with MMVD only (stage B) and seven healthy dogs without systemic or cardiovascular diseases.

### General characteristics

The baseline characteristics are summarized in Supplementary Table 1. The HAC + MMVD group consisted of six spayed females and one neutered male, including Pomeranians (n = 3), Toy Poodles (n = 2), Maltese (n = 1), and Chihuahua (n = 1). The MMVD stage was B1 in six dogs (6/7 dogs; 85.7%) and B2 in one dog (1/7 dogs; 14.3%). Noncardiac comorbidities included tracheal collapse (three dogs), chronic kidney disease IRIS stage 1 (two dogs), and other conditions (e.g., bronchial collapse, atlantoaxial band, gallbladder mucocele, hydrocephalus, and medial patellar luxation) in individual dogs.

No significant differences in age, sex, BCS, MBMI, WHSDR, WIWDR, or HR were observed between the HAC + MMVD and control groups. WTLR was significantly higher in the HAC + MMVD group than in the healthy control group (*p* = 0.017) but did not differ between the HAC + MMVD and MMVD only groups. SBP was significantly higher in the HAC + MMVD group than in the control group (*p* = 0.005 and *p* = 0.015, respectively). Systemic hypertension (SBP ≥ 150 mmHg) was observed in 3/7 dogs (42.9%) in the HAC + MMVD group, and in none of the controls. No significant differences in MMVD B stage severity or follow-up interval were between the HAC + MMVD and MMVD-only groups (*p* = 1.0 and *p* = 0.700, respectively).

### Clinicopathological findings

Tables 1 and 2 summarize the in-house and commercial laboratory results, respectively. The platelet count was significantly higher in the HAC + MMVD group than in the healthy (*p* = 0.017) and MMVD-only (*p* = 0.026) control groups. Potassium levels were significantly higher in the HAC + MMVD group than in the control group (*p* = 0.002 and *p* = 0.022, respectively). Plateletcrit, sodium, and D-dimer levels were significantly higher in the HAC + MMVD group than in either the MMVD-only control or healthy control groups.

**Table 1:** Descriptive statistics for in-house hematological and biochemical variables in the HAC + MMVD, health, and MMVD-only control groups

Variables	HAC + MMVD (n = 7)	Control group (n = 14)		Reference	*p* value
		Healthy (n = 7)	MMVD-only (n = 7)		
Platelets (K/uL)	428.0, 371–668	291.0, 150–446	342.0, 302–546	148–484	0.017^a,*^, 0.026^b,*^
MPV (fL)	12.4, 11.0–14.5	11.5, 10.6–13.6	12.3, 10.2–13.7	8.7–13.2	0.477^a^, 0.977^b^
PDW (fL)	13.8, 10.7–20.3	12.1, 8.5–14.5	12.4, 11.0–15.7	9.1–19.4	0.195^a^, 0.509^b^
Plateletcrit (%)	0.51, 0.47–0.82	0.35, 0.19–0.48	0.45, 0.38–0.56	0.14–0.46	0.003^a,**^, 0.058^b^
Glucose (mg/dL)	105.0, 88–133	102.0, 81–112	103.0, 95–149	70–143	0.302^a^, 0.780^b^
ALP (U/L)	148.0, 47–886	52.0, 29–179	60.0, 39–187	23–212	0.165^a^, 0.209^b^
Na+ (mmol/L)	150.0, 144–153	152.0, 150–163	155.0, 150–160	144–160	0.069^a^, 0.008^b,**^
K+ (mmol/L)	4.6, 4.2–5.2	4.1, 3.6–4.3	4.2, 3.5–4.6	3.5–5.8	0.002^a,**^, 0.022^b,*^
Cl− (mmol/L)	112.0, 109–123	114.0, 111–117	114.0, 111–121	109–122	0.307^a^, 0.425^b^
D-dimer (ng/dL)	173.46, 50.00–264.81	54.14, 50.00–81.85	115.66, 50.00–373.95	50–250	0.012^a,*^, 0.478^b^
Maximum amplitude	68.6, 57.2–79.4	65.9, 55.8–77.0	68.3, 47.4–72.3	42.9–67.9	0.535^a^, 0.456^b^
NT-proBNP (pmol/L)	1,043.6, 500.0–3,505.0	1,026.5, 500.0–1,856.1	638.5, 500.0–1,891.0	0–900	0.648^a^, 0.469^b^

Quantitative variables are expressed as the median, minimum-maximum.

Maximum amplitude is measured by thromboelastography.

HAC, hyperadrenocorticism; MMVD, myxomatous mitral valve disease; MPV, mean platelet volume; PDW, platelet distribution width; ALP, alkaline phosphatase; NT-proBNP, N-terminal pro B-type natriuretic peptide.

^a^*p* values were calculated using statistical comparisons between HAC + MMVD and healthy control group.

^b^*p* values were calculated using statistical comparisons between HAC+MMVD and MMVD-only control group.

^*^p < 0.05, ^**^p < 0.01.

**Table 2:** Descriptive statistics for commercial laboratory hematological and biochemical variables in the HAC + MMVD, healthy, and MMVD-only control groups

Variables	HAC + MMVD (n = 7)	Control group (n = 14)		Reference	*p* value
		Healthy (n = 7)	MMVD-only (n = 7)		
Urinary Normetanephrine:creatinine	495.2, 167.2–990.5	149.8, 72.5–297.4	163.8, 77.3–264.9	N/A	0.011^a,*^, 0.007^b,**^
Urinary Metanephrine:creatinine	100.0, 61.4–760.0	66.7, 43.2–130.1	121.5, 24.6–213.6	N/A	0.053^a^, 0.620^b^
TCHOL (mg/dL)	263.0, 162–412	235.0, 135–305	127.0, 108–233	131–345	0.383^a^, 0.026^b*^
HDL-C (mg/dL)	176.0, 130–312	176.0, 111–234	100.0, 74–192	97–173	0.555^a^, 0.091^b^
LDL-C (mg/dL)	57.0, 11–87	38.0, 14–59	17.0, 14–38	N/A	0.430^a^, 0.220^b^
TG (mg/dL)	100.0, 60–133	61.0, 42–85	70, 40–75	20–150	0.026^a,*^, 0.034^b,*^
TC:HDL-C	1.321, 1.224–1.494	1.234, 1.195–1.339	1.270, 1.214–1.459	N/A	0.118^a^, 0.364^b^
TG:HDL-C	0.520, 0.215–0.808	0.372, 0.261–0.535	0.400, 0.313–0.986	N/A	0.119^a^, 0.736^b^

Quantitative variables are expressed as the median, minimum-maximum.

HAC, hyperadrenocorticism; MMVD, myxomatous mitral valve disease; N/A not applicable; TCHOL, total cholesterol; HDL-C, High-density lipoprotein cholesterol; LDL-C, Low-density lipoprotein cholesterol; TG, triglyceride; TC:HDL-C, Total cholesterol-to-HDL-C ratio; TG:HDL-C, Triglyceride-to-HDL-C ratio.

^a^*p* values were calculated using statistical comparisons between HAC + MMVD and healthy control group.

^b^*p* values were calculated using statistical comparisons between HAC + MMVD and MMVD-only control group.

^*^p < 0.05, ^**^p < 0.01.

The urinary normetanephrine: creatinine ratio was significantly higher in the HAC + MMVD group than in the healthy (*p* = 0.011) and MMVD-only (*p* = 0.007) control groups. Triglyceride levels were also significantly higher in the HAC + MMVD group than in the healthy and MMVD-only control groups (*p* = 0.026 and *p* = 0.034, respectively). Total cholesterol levels were significantly higher in the HAC + MMVD group than in the MMVD only control group (*p* = 0.026).

### Echocardiographic findings

#### Conventional echocardiography

Table 3 presents conventional echocardiographic data. The LVFWdN and IVSdN were significantly higher in the HAC + MMVD group than in the healthy and MMVD-only control groups. In contrast, LA:Ao, LVIDdN, LVIDsN, and mitral E/A ratio were significantly lower in the HAC + MMVD group than in the healthy and MMVD-only control groups. Additionally, IVSsN, E/E′, and EF were significantly higher in the HAC + MMVD group than in the healthy control group.

**Table 3:** Conventional echocardiographic variables of the HAC + MMVD, healthy, and MMVD-only control groups

Variables	HAC + MMVD (n = 7)	Control groups (n = 14)		*p* value
		Healthy (n = 7)	MMVD-only (n = 7)	
LA:Ao ratio	1.07, 1.01–1.22	1.26, 1.10–1.30	1.27, 1.11–1.73	0.038^a,*^, 0.038^b,*^
LVIDdN	0.862, 0.748–1.039	1.360, 1.035–1.683	1.517, 1.065–1.689	0.001^a,**^, 0.001^b,**^
LVIDsN	0.390, 0.210–0.454	0.849, 0.444–1.126	0.601, 0.437–0.879	0.002^a,**^, 0.002^b,**^
LVFWdN	0.641, 0.576–0.690	0.436, 0.348–0.511	0.391, 0.312–0.497	0.001^a,**^, 0.001^b,**^
LVFWsN	0.855, 0.721–1.009	0.719, 0.648–0.945	0.734, 0.554–0.919	0.165^a^, 0.097^b^
IVSdN	0.611, 0.477–0.726	0.438, 0.346–0.489	0.508, 0.381–0.539	0.002^a,**^, 0.007^b,**^
IVSsN	0.798, 0.698–0.958	0.643, 0.507–0.797	0.716, 0.655–0.907	0.004^a,**^, 0.128^b^
E vel (cm/s)	65.67, 44.70–79.55	56.58, 53.86–65.51	65.75, 49.51–77.69	0.209^a^, 0.805^b^
A vel (cm/s)	91.41, 56.76–136.60	59.63, 44.37–96.82	66.63, 58.74–80.06	0.073^a^, 0.097^b^
Mitral E/A ratio	0.70, 0.51–0.85	0.95, 0.64–1.32	0.98, 0.71–1.19	0.038^a,*^, 0.007^b,**^
E/E‘	16.06, 11.74–20.33	10.19, 7.17–14.76	12.88, 6.50–16.23	0.011^a,*^, 0.165^b^
FS (%)	59.5, 38.4–75.3	45.2, 28.0–63.8	56.1, 41.5–68.57	0.053^a^, 0.535^b^
EF (%)	91.7, 73.6–98.0	78.9, 53.97–92.93	82.9, 63.67–95.13	0.026^a,*^, 0.209^b^
Tei index	0.1, −0.2–0.7	0.2, −0.7–0.3	0.1, −0.4–0.2	0.972^a^, 0.481^b^
IVRT (ms)	54.67, 39.67–80.00	83.00, 41.00–156.67	61.33, 27.67–112.23	0.128^a^, 0.710^b^
E/IVRT	1.09, 0.760–1.660	0.80, 0.37–1.83	0.99, 0.70–2.70	0.272^a^, 0.871^b^

Quantitative variables are expressed as the median, minimum-maximum. Normalizing M-mode variables using body weight were calculated according to the regression equation reported by Cornell et al. [13].

HAC, hyperadrenocorticism; MMVD, myxomatous mitral valve disease; LA, left atrium; Ao, aortic root diameter; LVIDdN, normalized left-ventricular internal diameter in diastole, LVIDsN, normalized left-ventricular internal diameter in systole; LVFWdN, normalized left-ventricular free-wall thickness in diastole; LVFWsN, normalized left-ventricular free-wall thickness in systole; IVSdN, normalized interventricular septum thickness in diastole; IVSsN, normalized interventricular septum thickness in systole; E vel, early diastolic wave velocity of transmitral flow; A vel, late diastolic wave velocity of transmitral flow; E’, early diastolic myocardial velocity on tissue doppler imaging; FS, fractional shortening; EF, ejection fraction; IVRT, isovolumetric relaxation time.

^a^*p* values were calculated using statistical comparisons between HAC + MMVD and healthy control group.

^b^*p* values were calculated using statistical comparisons between HAC + MMVD and MMVD-only control group.

^*^p < 0.05, ^**^p < 0.01.

#### STE

Supplementary Table 2 summarizes the 2D-STE findings. GLS was significantly reduced in the HAC + MMVD group compared to both the healthy (*p* = 0.001) and MMVD-only (*p* = 0.002) control groups. Segmental LS values were significantly reduced in the HAC + MMVD group compared with those in the healthy control group (*p* < 0.05, for all segments except the basal lateral segment; *p* = 0.073) but not with the MMVD-only control group. GLS demonstrated acceptable intra- and inter-observer reproducibility (CV < 15%).

### Associations with left ventricular wall thickness in the HAC + MMVD group

#### Signalment

No significant associations between LV wall thickness (LVFWdN and IVSdN) and age, sex, or breed were observed.

#### Physical and laboratory variables

IVSdN was significantly correlated with the WIWDR (r = 0.857, *p* = 0.014), platelet count (r = 0.857, *p* = 0.014), plateletcrit (r = 0.929, *p* = 0.003), and urinary normetanephrine: creatinine ratio (r = 0.857, *p* = 0.014). The LVFWdN correlated with total cholesterol (r = 0.857, *p* = 0.014). No other significant associations were observed (Table 4).

**Table 4:** Correlation coefficients between the cardiac wall thickness (LVFWdN and IVSdN) and physical examination and cardiovascular variables in dogs with HAC + MMVD

Variables	LVFWdN	IVSdN	GLS
WHSDR	0.036 (0.939)	0.643 (0.119)	−0.536 (0.215)
WIWDR	0.214 (0.645)	0.857 (0.014)^*^	−0.198 (0.670)
WTLR	−0.429 (0.337)	0.036 (0.939)	−0.571 (0.180)
SBP	0.270 (0.558)	−0.739 (0.058)	−0.036 (0.939)
Platelets	0.071 (0.879)	0.857 (0.014)^*^	0.214 (0.645)
Plateletcrit	−0.179 (0.702)	0.929 (0.003)^**^	0.286 (0.535)
Na+	−0.074 (0.875)	−0.037 (0.937)	0.630 (0.129)
K+	−0.071 (0.879)	0.179 (0.702)	−0.071 (0.879)
d-dimer	0.071 (0.879)	0.071 (0.879)	0.071 (0.879)
Urinary normetanephrine:creatinine	0.357 (0.432)	0.857 (0.014)^*^	0.179 (0.702)
TCHOL	0.857 (0.014)^*^	0.214 (0.645)	0.464 (0.294)
TG	0.000 (1.000)	0.464 (0.294)	−0.036 (0.939)

In the table, coefficient values are described as rho (*p* value).

LVFWdN, normalized left-ventricular free-wall thickness in diastole; IVSdN, normalized interventricular septum thickness in diastole; HAC, hyperadrenocorticism; MMVD, myxomatous mitral valve disease; GLS, global longitudinal strain; WHSDR, waist-to-hock-to-stifle distance ratio; WIWDR, waist-to-ilium wing distance ratio; WTLR, waist-to-truncal length ratio; SBP, systemic blood pressure; TCHOL, total cholesterol; TG, triglyceride.

^*^p < 0.05, ^**^p < 0.01.

#### Multivariable analysis

Multivariable linear regression showed urinary normetanephrine: creatinine ratio as the sole independent predictor of IVSdN (B = 0.000222, 95% confidence interval [CI], 0.000050 to 0.000393, *p* = 0.026) among platelet count, plateletcrit, and urinary normetanephrine: creatinine ratio.

### Associations with left ventricular systolic function

GLS was not significantly associated with any of the variables, including urinary normetanephrine: creatinine ratio (rho = 0.179, 95% CI, −0.698 to 1.000, *p* = 0.720, Table 4).

### Longitudinal changes from baseline to re-evaluation

#### Follow-up population and physical examination

Seven dogs in the HAC + MMVD group and six dogs in the MMVD only group underwent follow-up. In the MMVD control group, one dog was excluded because the owner was lost to follow-up. In the study group, at re-evaluation, one dog’s owner did not provide a urine sample; therefore, urine-based analyses were performed on six dogs only.

The reevaluation interval between inclusion (T1) and reevaluation (T2) did not differ between the HAC + MMVD and MMVD-only control groups (*p* = 0.700). No significant differences were seen at T2 or in Δ (T2−T1) for BW, MBMI, anthropometric ratios, HR, or SBP (Supplementary Table 3).

#### Biochemical changes

No significant differences were observed between groups at re-evaluation (T2) or in Δ values for urinary normetanephrine: creatinine, urinary metanephrine: creatinine, TCHOL, HDL-C, LDL-C, TG, TC:HDL-C, and TG:HDL-C (Supplementary Table 4).

#### Echocardiographic changes

At T2, the HAC + MMVD group had significantly lower LVIDdN, LVIDsN, and E/A ratios (*p* = 0.035, 0.014, and 0.022, respectively) and significantly higher IVSdN (*p* = 0.008) than the MMVD-only group. LVFWdN showed a trend (*p* = 0.051). No other significant between-group differences in conventional echocardiographic variables were observed at T2. Between-group comparisons of change scores (Δ = T2−T1) were not significant for any variable. At T2, the GLS was higher in MMVD-only group than in HAC+MMVD group (*p* = 0.001). No significant Δ differences were observed between groups (Supplementary Table 5).

#### Associations with LV wall thickness

Δ Urinary normetanephrine: creatinine was positively associated with Δ IVSdN after adjusting for Δ days (B = 0.000308, 95% CI, 0.000108 to 0.000507, *p* = 0.016). To visualize the relationship between Δ urinary normetanephrine: creatinine and Δ IVSdN, a partial regression (added-variable) plot adjusted for Δ days was generated and the association was also significantly positive (B = 0.000308, *p* = 0.005; Fig. 2A). The actual and predicted values showed close agreement along the 45° line, consistent with a strong overall model fit (*p* = 0.029; Fig. 2B). Residuals versus predicted values were centered at approximately zero with no evident curvature or funneling, indicating no gross violations of linearity or homoscedasticity (Fig. 2C).

**Fig. 2:**
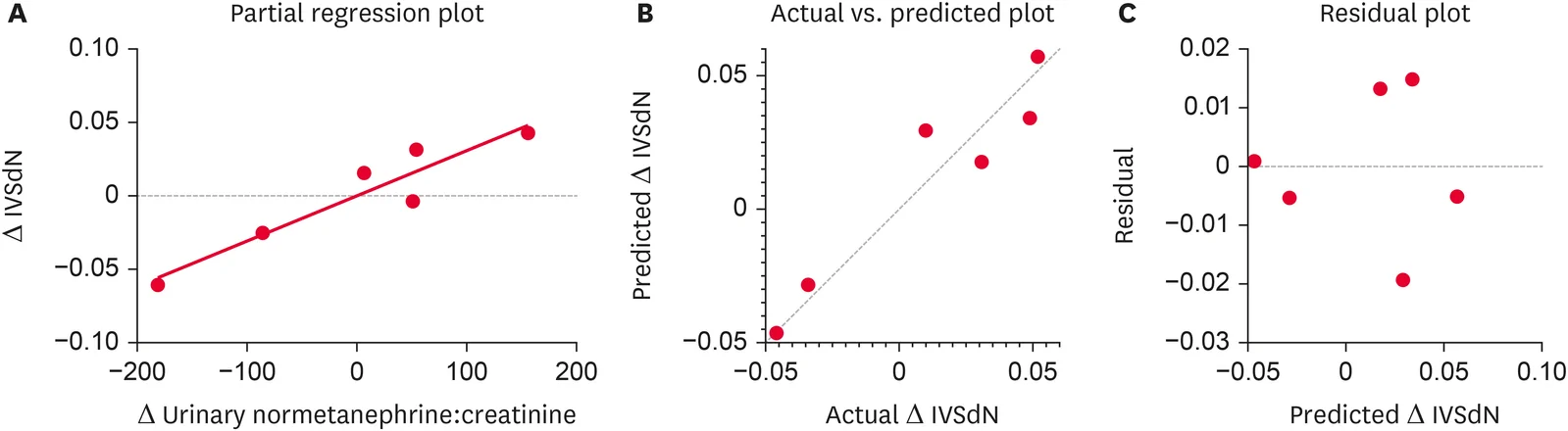
Model diagnostics and adjusted association between Δ urinary normetanephrine:creatinine and Δ IVSdN. (A) Partial regression (added-variable) plot adjusted for Δ days. Δ urinary normetanephrine: creatinine was significantly positively associated with ΔIVSdN (B = 0.000308, *p* = 0.005) (B) Actual vs. Predicted ΔIVSdN. Points close to the 45° line indicate good agreement between observed and fitted values. (C) Residuals vs. Predicted Δ IVSdN. Residuals are centered around zero without an obvious pattern. Predicted values and residuals are from a multiple linear regression with Δ urinary normetanephrine:creatinine as the primary predictor; Δ days was included only as a covariate. In this model, Δ normetanephrine was a significant predictor of Δ IVSdN (B = 0.000308, 95% confidence interval 0.000108–0.000507, *p* = 0.016), and overall model fit was R^2^ = 0.9057 (F(2,3) = 14.40, *p* = 0.029). IVSdN, normalized interventricular septum thickness in diastole.

Δ Platelet count and Δ plateletcrit were not significantly associated with Δ IVSdN after adjusting for Δ days (*p* = 0.282 and *p* = 0.400, respectively). Similarly, Δ total cholesterol was not significantly associated with Δ LVFWdN after adjusting for Δ days (*p* = 0.589).

#### Associations with LV systolic function

Multiple regression adjusting for Δ days showed no significant associations between ΔGLS and Δ normetanephrine (*p* = 0.453) or Δ total cholesterol (*p* = 0.535).

## DISCUSSION

HAC induces cardiovascular alterations through multiple pathophysiological mechanisms, including LVH and myocardial dysfunction [3,5,6,27]. In humans, these changes have been extensively studied in the context of endogenous Cushing’s syndrome and pheochromocytoma. However, in veterinary medicine, evidence regarding the pathophysiological contribution of HAC to cardiac remodeling, especially in dogs with concurrent MMVD, remains limited.

In this prospective pilot study, the urinary normetanephrine: creatinine ratio was significantly higher in dogs with HAC + MMVD than in both healthy and MMVD-only control dogs, which is consistent with previous findings in dogs with HAC [19,28]. Furthermore, the urinary normetanephrine: creatinine ratio was significantly associated with increased IVS thickness at baseline and over time (Δ IVSdN), suggesting that catecholamine excess may play a role in promoting myocardial hypertrophy [18,29,30,31].

HAC may also increase circulating catecholamines through cortisol-mediated enzymatic upregulation (e.g., tyrosine hydroxylase, phenylethanolamine N-methyltransferase, and dopamine β-hydroxylase), enhancing sympathetic tone and adrenal medullary output [20,32]. These endocrine effects may explain the observed ventricular remodeling in the absence of persistent systemic hypertension, which was not associated with myocardial thickness in this study, confirming the results of prior studies that also failed to link HAC-associated hypertension to hypertrophy [13,33,34].

Metabolic abnormalities, such as elevated triglyceride and total cholesterol levels, were observed in the HAC + MMVD group, in agreement with previously reported dyslipidemia in HAC [4]. Of these variables, only total cholesterol level was significantly correlated with LVFW thickness at baseline. Although no longitudinal association (Δ total cholesterol vs. Δ LVFWdN) was found, the possibility of cholesterol-driven hypertrophic mechanisms—such as lipid accumulation and oxidative stress—remains biologically plausible [17,35,36].

Platelet count and plateletcrit were also significantly higher in the HAC + MMVD group, and both correlated with IVSdN at baseline, although they did not predict Δ IVSdN. A previous study in humans suggested a link between platelet activation and myocardial hypertrophy, but typically not with the platelet count itself [37]. Therefore, these findings may reflect the indirect effects of hypercortisolemia rather than direct hemodynamic drivers of hypertrophy.

Conventional echocardiographic indices, such as EF and FS, did not consistently differentiate the HAC + MMVD group from the MMVD-only control group, whereas STE-derived GLS was significantly impaired in dogs with HAC + MMVD. These results are consistent with those of previous studies [1,2]. However, GLS was not significantly associated with normetanephrine or lipid profiles in this study, possibly because the small sample size limited the statistical power to detect such associations, and because the results may also have been confounded by MMVD. This may indicate that although normetanephrine was associated with myocardial thickening, the degree of catecholamine-related structural remodeling was not sufficient to produce measurable functional impairment. In addition, functional changes may be influenced by multiple factors such as increased afterload related to elevated wall stress, myocardial ischemia, and desynchrony.

Importantly, several echocardiographic indices or laboratory variables (e.g., E/E′, and EF, plateletcrit, D-dimer) differed from that of healthy controls but not from that of MMVD-only controls, suggesting that these changes may represent MMVD-related alterations rather than HAC-specific effects. The inclusion of the MMVD-only control group was essential to distinguish additive endocrine contributions from the cardiac phenotype alone.

In the present study, the HAC + MMVD group had smaller LA:Ao and LVIDdN values than both the healthy control and MMVD-only control groups, despite the eccentric remodeling typically expected in MMVD. One possible explanation is that HAC may have promoted concentric hypertrophy, thereby reducing the apparent chamber dimensions. In addition, these findings may be influenced by the small sample size and individual variability among dogs, although the groups were matched within MMVD stage B.

This study had several limitations. First, the small sample size limited the statistical power. As this study was designed as an exploratory pilot study, we initially planned to enroll at least 12 dogs per group [38]; however, only 7 dogs per group were finally included. This was primarily due to the rarity of dogs meeting all predefined eligibility criteria within the limited study period, particularly given the rigorous inclusion and exclusion criteria and the longitudinal design of the study. Therefore, the findings should be interpreted as primarily identifying trends rather than providing definitive conclusions. Second, although pheochromocytomas could not be definitively excluded without histopathological confirmation, clinical signs and abdominal ultrasound findings were unlikely. Third, a single large-breed dog (Golden Retriever) in the healthy control group skewed the body weight comparisons, although normalization of wall thickness indices minimized its influence. Fourth, the absence of an HAC-only group limited our ability to distinguish the effects of HAC itself from those related to the interaction between HAC and MMVD. It also remains unclear whether HAC-related corticosteroid excess directly induced the observed changes or whether these effects were mediated indirectly through the cardiovascular system. Therefore, the observed cardiac and biomarker changes should be interpreted as findings associated with concurrent HAC in dogs with MMVD, rather than as effects attributable solely to HAC.

To our knowledge, this is the first prospective study to investigate the potential mechanisms underlying LVH and dysfunction in dogs with concurrent HAC and MMVD. Our findings suggest that elevated catecholamine levels, particularly urinary normetanephrine levels, may be associated with IVS thickening in this population, paralleling those of human pheochromocytomas studies and implicating sympathetic overactivity in cardiac remodeling.

Although conventional systolic function indices did not differ consistently between the groups, GLS was significantly impaired in the HAC + MMVD group, highlighting the value of STE in detecting subtle myocardial dysfunction. Additionally, total cholesterol and platelet-related parameters were associated with baseline wall thickness, although their longitudinal influence was limited.

In conclusion, these findings suggest that catecholamines may be candidate biomarkers of ventricular remodeling in dogs with HAC and highlight the need for further evaluation of metabolic contributors. The plausible endocrine–cardiac interactions identified in this exploratory pilot study provide a basis for future investigations in both veterinary and comparative cardiology. Larger, multicenter longitudinal studies are warranted to validate these observations and clarify the underlying mechanistic pathways.

## SUPPLEMENTARY MATERIALS

Supplementary Table 1 General characteristics of the HAC + MMVD, health, and MMVD-only control groups

Supplementary Table 2 Variables of left ventricle longitudinal peak strains (LS) derived from two-dimensional speckle-tracking echocardiography in the HAC + MMVD, healthy, and MMVD-only control groups

Supplementary Table 3 Physical examinations between inclusion and re-evaluation of the HAC + MMVD and MMVD-only control group

Supplementary Table 4 Biochemical variables between inclusion and re-evaluation of the HAC + MMVD and MMVD-only control group

Supplementary Table 5 Echocardiographic variables between inclusion and re-evaluation of the HAC + MMVD and MMVD-only control group
